# Validation of Novel Microsurgical Vessel Anastomosis Techniques: A Systematic Review

**DOI:** 10.1055/a-2302-7126

**Published:** 2024-05-06

**Authors:** Yasmin Sadigh, Imen Mechri, Anamika Jain, Amata Thongphetsavong Gautam, Hadil Seh, Victor Volovici

**Affiliations:** 1 Department of Neurosurgery, Erasmus MC Stroke Center, Erasmus MC University Medical Centre, Rotterdam, The Netherlands; 2 Department of Neurosurgery, University of Medicine and Pharmacy “Grigore T. Popa”, Iasi, Romania; 3 National Department of Neurosurgery, Centre Hospitalier de Luxembourg, Luxembourg, Luxembourg; 4 Department of Neurosurgery, Soroka Medical Center, Beer Sheva, Israel; 5 Department of Public Health, Centre for Medical Decision Science, Erasmus MC University Medical Centre, Rotterdam, The Netherlands

**Keywords:** microsurgery, surgical technique, vessel anastomosis, validation

## Abstract

**Background** Thorough validation of novel microsurgical techniques is deemed essential before their integration into clinical practice. To achieve proper validation, the design of randomized controlled trials (RCTs) should be undertaken, accompanied by the execution of comprehensive statistical analyses, including confounder adjustment and power analysis. This systematic review aims to provide an encompassing overview of the validation methodologies employed in microsurgical studies, with a specific focus on innovative vessel anastomosis techniques.

**Methods** A literature search was conducted in PubMed for articles describing the validation of novel microsurgical vessel anastomosis techniques in animal or human subjects.

**Results** The literature search yielded 6,658 articles. A total of 6,564 articles were excluded based on title and abstract. Ninety-four articles were assessed for full-text eligibility. Forty-eight articles were included in this systematic review. Out of 30 comparative studies, 9 studies validated novel modified interrupted suture techniques, 6 studies modified continuous techniques, 6 studies modified sleeve anastomosis techniques, 1 study a modified vesselotomy technique, 7 studies sutureless techniques, and 1 study a modified lymphaticovenular anastomosis technique. Twenty-eight studies contained animals ( *n* = 1,998). Fifteen animal studies were RCTs. Two studies contained human/cadaveric subjects ( *n* = 29). Statistical power analysis and confounder adjustment were performed in one animal study. Out of 18 noncomparative studies, 5 studies validated novel modified interrupted suture techniques, 1 study a modified continuous technique, 2 studies modified sleeve anastomosis techniques, 4 studies modified vesselotomy techniques, 4 studies sutureless techniques, and 2 studies modified lymphaticovenular anastomosis techniques. Ten studies contained animal subjects ( *n* = 320), with two RCTs. Eight studies contained human subjects ( *n* = 173). Statistical power analysis and confounder adjustment were performed in none of the animal or human studies.

**Conclusion** The current methods of microsurgical technique validation should be reconsidered due to poor study design. Statistical analysis including confounder adjustment and power analysis should be performed as a standard method of novel technique validation.

Microsurgery is a complex and precise field of surgery, which requires a high level of technical skills. 1 Microsurgical techniques are used in a wide variety of surgical subspecialties, for example, vessel and nerve anastomosis, which allows for repair of human tissue and regain of function after trauma, tumor resection, anatomical reconstructions, congenital abnormalities, and impending ischemia ( Fig. 1A and B ). 1 To construct patent microsurgical vessel anastomoses in patients needing complex vascular reconstructions using new and improved techniques, these techniques need to be validated. Proving the validity of a new microsurgical technique could predict their safe and effective use in the clinical practice. Designing randomized controlled trials (RCTs), statistical analysis and confounder adjustment involving outcome measures such as anastomosis time and patency rate are necessary to be able to grant validation to new microsurgical techniques. In the past decades, over 2,000 articles on microsurgical anastomosis techniques have been published. 2 This systematic review aims to provide an overview of validation methods used by microsurgical studies, proposing new vessel anastomosis techniques.

**Fig. 1:**
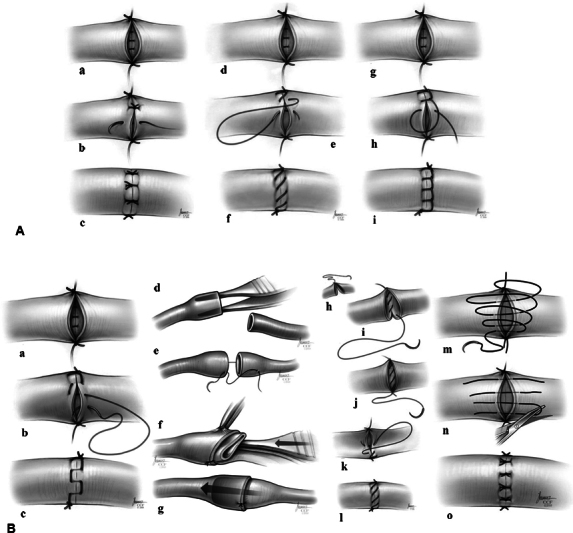
( **A** ) Schematic illustration of the simple interrupted technique. The vessel ends are bisected with two stay sutures placed at 180 degrees (the posterior wall is finished) ( **a** ). The suture is passed full thickness from the outside-in direction of one vessel end into the lumen and then from the inside-out through the other vessel end ( **b** ). The knot is tied on the outside and step B is repeated finishing the anterior wall ( **c** ). Schematic illustration of the continuous suture technique. The vessel ends are bisected with two stay sutures and one end is used to finish the posterior wall after flipping the clamps ( **d** ). Each suture is passed from the outside of the donor vessel to the inside of the recipient vessel ( **e** ). A total of two knots are tied at the end of the procedure: one at the apex and one at the base ( **f** ). Schematic illustration of continuous locking technique. The vessel ends are bisected with two stay sutures and one end is used to finish the posterior wall after flipping the clamps ( **g** ). Each suture is passed from the outside of the donor vessel to the inside of the recipient vessel and locked after each pass ( **h** ). A total of two knots are tied at the end of the procedure: one at the apex and one at the base ( **i** ). Reproduced with permission from John Wiley and Sons (January 20, 2024) from Alghoul MS, Gordon CR, Yetman R, Buncke GM, Siemionow M, Afifi AM, Moon WK. From simple interrupted to complex spiral: a systematic review of various suture techniques for microvascular anastomoses. Microsurgery 2011;31(1):72–80. ( **B** ) Schematic illustration of continuous horizontal mattress technique. The first step is similar to the conventional continuous suture in ( **a** ). Each suture is passed from the outside of the donor vessel to the inside of the recipient vessel, the needle's direction is then reversed to allow placement of a horizontal mattress fashion ( **b** ), which is then continued in an uninterrupted fashion around the entire suture line ( **c** ). Schematic illustration of the sleeve anastomosis. Sufficient gentle dilatation of the proximal (feeding) vessel end ( **d** ). Partial thickness bites (without entering the vessel lumen) placed at a distance approximately one and half times the vessel diameter from the vessel and passed through the inner side of the distal vessel end in an inside-out fashion and then tied ( **e** ). The proximal folded vessel is gently tucked inside the distal vessel with another forceps ( **f** ). Completed anastomosis ( **g** ). Schematic illustration of Timmon's modified continuous suture technique. After the first knot is tied ( **h** ), a short remnant is left on one end and the other end is ran continuously to suture closed the posterior wall. The suture is then pulled snug with each pass instead of keeping the edges separated until the end. After the posterior wall is complete, the suture is cut leaving behind a short remnant ( **i** ). A second knot is tied (180 degrees to the first knot) using a second suture, and the remnant is tied to the second knot ( **j** ). The suture is then ran along the anterior wall ( **k** ) and tied to the first suture remnant completing the anastomosis ( **l** ). Schematic illustration of the spiral anastomosis. A loose running suture is placed to form a decrescendo spiral (loops) on the surface of the anastomosis ( **m** ). This suture then becomes interrupted following tangential cuts made through the loops ( **n** ). All suture segments are then tied individually as similar to the common interrupted technique ( **o** ). Reproduced with permission from John Wiley and Sons (January 20, 2024) from Alghoul MS, Gordon CR, Yetman R, Buncke GM, Siemionow M, Afifi AM, Moon WK. From simple interrupted to complex spiral: a systematic review of various suture techniques for microvascular anastomoses. Microsurgery 2011;31(1):72–80.

## Methods

A literature search was conducted in PubMed from database inception to January 6, 2024. Combinations of search terms regarding “Microsurgical techniques” were used. To be included, articles had to meet the following criteria: (1) microsurgical techniques had to be applied to blood vessel anastomosis, (2) studies had to include human or animal subjects, (3) articles had to contain more than 5 subjects, and (4) articles must be written in English. Reasons for exclusion of articles were (1) nonoriginal articles, (2) articles reporting data on other types of anastomoses than blood vessels, (3) reviews, and (4) training models.

The title and abstract of the articles were screened for eligibility by five of the authors (Y.S., I.M., A.J., A.T.G., H.S.). If their abstracts met the inclusion criteria, full-text articles were obtained. Each full-text article was then assessed for final inclusion in this systematic review. All articles were rescreened and reassessed by one author (Y.S.). Disagreements were resolved through discussion with the senior author (V.V.).

Relevant data from included articles were extracted by independent authors (Y.S., I.M., A.J., A.T.G., H.S.) into an extraction template. Study information extracted included: study design (comparative, noncomparative, RCT, experimental, retrospective observational cohort, case series), country, number of study subjects and type of subjects (animal, human), type of novel modification, type of anastomosis (end-to-end, end-to-side, side-to-side), description of technique, the study's main outcome(s), control group, if technique is clinically used, whether power analysis was performed, and whether confounder adjustment was performed. All data was reextracted by one author (Y.S.). The novel microsurgical techniques were divided into six groups: modified interrupted suture techniques, modified continuous techniques, modified sleeve anastomosis techniques, modified vesselotomy techniques, sutureless techniques, and modified lymphaticovenular anastomosis techniques.

Quality assessment of the included articles was performed by the senior author (V.V.) using Cochrane RCT-2 for RCTs and A Cochrane Risk Of Bias Assessment Tool: for Non-Randomized Studies of Interventions (ACROBAT-NRSI) for nonrandomized studies.

## Results

**Fig. 2:**
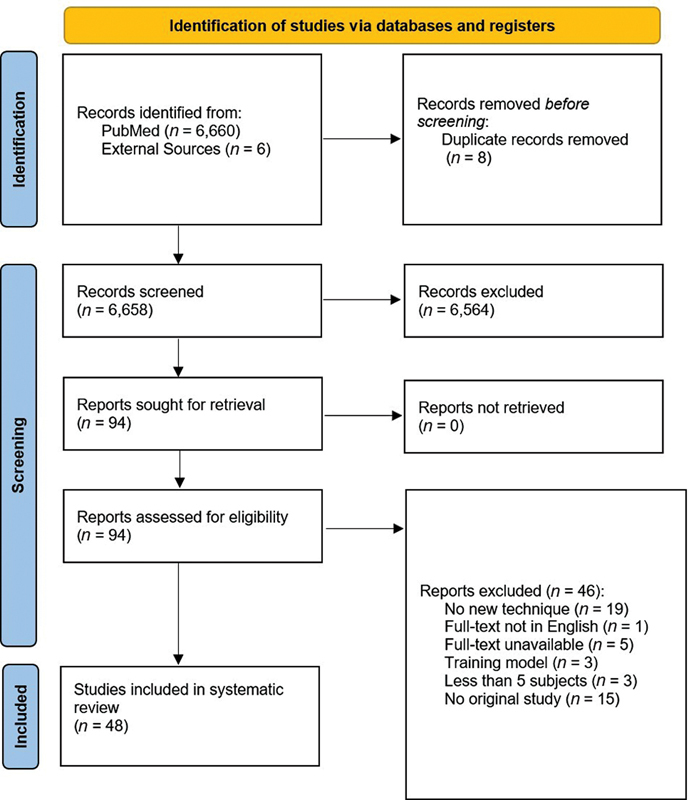
Flowchart of literature search. Page MJ, McKenzie JE, Bossuyt PM, Boutron I, Hoffmann TC, Mulrow CD, et al. The PRISMA 2020 statement: an updated guideline for reporting systematic reviews. BMJ 2021;372:n71.

The search strategy yielded 6,658 articles, which were put through initial screening. A total of 6,564 articles were excluded based on title and abstract. Ninety-four articles remained to be assessed for eligibility based on full-text sifting. Ultimately, 48 articles were included in this systematic review based on full-text eligibility ( Fig. 2 ). Quality assessment revealed a low risk of bias for RCTs ( *n* = 17), and for the nonrandomized studies ( *n* = 31) a critical risk of bias.

### Comparative Studies

Thirty of the included studies were comparative studies ( Table 1 ). In total, 28 studies contained animal subjects ( *n* = 1,998 animal subjects) and two studies contained human subjects ( *n* = 29) ( Supplementary Appendix: Table S1 , available in the online version).

**Table 1:** Studies validating novel microsurgical techniques: comparative studies (30 studies)

Technique type	Study design (no. studies)	Type of subjects (no. studies)	Type of modification (no. studies)	Anastomosis type (no. studies)	Main outcome (no. studies)	Control group (no. studies)	Technique is clinically used (no. studies)	Power analysis performed (no. studies)	Confounder adjustment performed (no. studies)
Modified interrupted technique (9 studies) 3 11 12 14 19 20 21 22 23	Comparative (3) RCT (5) RO (1)	Animal (7) Human (2)	Posterior wall first (1) Posterior wall support (1) Fewer sutures + glue (1) Extra cut placement (1) Flow-through anastomosis (1) Antegrade anastomosis (1) Intravascular stent (1) Double stitch everting (2) Horizontal mattress sutures (1)	ETE (6) ETS (7)	Postoperative PR (4) Postoperative FR (3) Flap survival (1) Ischemic time (1) Suture symmetry score (1) Anastomotic leakage (1) Anastomotic time (1) Thrombosis rate (1)	Conventional interrupted suture technique (9)	Yes (2) No (2) N/A (5)	Yes (1) No (8)	Yes (1) No (8)
Modified continuous technique (6 studies) 10 15 17 18 24 25	Experimental (2) RCT (4)	Animal (6)	Posterior wall first (1) Absorbable sutures (1) Horizontal mattress sutures (1) Knotless (2) Inside-to-outside suture (1)	ETE (4) ETS (2)	Postoperative PR (2) Postoperative FR (2) Anastomotic leakage (1) Aneurysm formation (1) Postoperative anastomosis bleeding and stenosis (1) Graft and subject survival (1)	Conventional continuous suture technique (4) Conventional interrupted suture technique (3)	Yes (1) N/A (6)	No (6)	No (6)
Modified sleeve anastomosis technique (6 studies) 8 9 26 27 28 29	Comparative (1) Experimental (3) RCT (2)	Animal (6)	Asymmetrical sleeve (1) Symmetrical sleeve (1) Extra cut placement (2) Suture placement (3) Glue (1)	ETE (6)	Postoperative PR (5) Elongation, tensile strength, and elasticity of vessels (1)	Conventional interrupted suture technique (5) Conventional interrupted suture technique (1) Arteries with adventitia (1)	No (1) N/A (5)	No (6)	No (6)
Modified vesselotomy technique (1 study) 4	RCT (1)	Animal (1)	Elliptic vesselotomy (1)	ETS (1)	Postoperative PR (1)	Slit anastomosis in arteries and veins (1)	Yes (1)	No (1)	No (1)
Sutureless technique (7 studies) 13 16 28 30 31 32 33	Comparative (2) Experimental (3) RCT (2)	Animal (7)	Photochemical bonding (1) Absorbable cuff (1) Unabsorbable cuff (1) Glue + absorbable stent (1) Glue + venous cuff (1) Biodegradable laser (1) Glue (1)	ETE (7)	Postoperative PR (4) Postoperative FR (1) Cuff absorption rate (1) Surgical success rate (1) Anastomotic time (2)	Conventional interrupted suture technique (6) Unabsorbable cuff technique (1)	Yes (1) No (1) N/A (5)	No (7)	No (7)
Modified lymphaticovenular anastomosis technique (1 study) 5	RCT (1)	Animal (1)	Intima-to-intima coaptation (1)	ETE (1)	Postoperative PR (1)	Conventional lymphaticovenular implantation technique (1)	No (1)	No (1)	No (1)

Abbreviations: ETE, end-to-end; ETS, end-to-side; FR, flow rate; N/A, not available; PR, patency rate; RCT, randomized controlled trial; RO, retrospective observational.

Nine studies proposed a novel modified interrupted anastomosis technique, with modifications such as double stitch everting in two studies ( Table 1 ). Seven studies were performed on animal subjects and two on human subjects ( Table 1 ). Five studies were RCTs ( Table 1 ). Conventional interrupted suture technique was used in the control group in all nine studies ( Table 1 ). The immediate postoperative patency rate ( *n* = 4) and the postoperative flow rate ( *n* = 3) were the most investigated main outcomes ( Table 1 ). Two techniques were clinically used, and for five techniques details on their clinical use was unavailable ( Table 1 ). Statistical power analysis and confounder adjustment were performed in one animal study by Dindelegan et al 3 ( Table 1 ).

Six studies proposed a novel modified continuous anastomosis technique, with modifications such as knotless continuous anastomosis in two studies ( Table 1 ). All studies were performed on animal subjects ( Table 1 ). Four studies were RCTs ( Table 1 ). Conventional interrupted suture technique was used in the control group in three studies and conventional continuous suture technique in four studies ( Table 1 ). The immediate postoperative patency rate ( *n* = 2) and the postoperative flow rate ( *n* = 2) were the most investigated main outcomes ( Table 1 ). Two techniques were clinically used, and for five techniques details on their clinical use was unavailable ( Table 1 ). Statistical power analysis and confounder adjustment were performed in none of the studies ( Table 1 ).

Six studies proposed a novel modified sleeve anastomosis technique, with modifications such as suture placement in three studies and extra cut placement in two studies ( Table 1 ). All studies were performed on animal subjects ( Table 1 ). Two studies were RCTs ( Table 1 ). Conventional interrupted suture technique was used in the control group in five studies and conventional continuous suture technique in one study ( Table 1 ). The immediate postoperative patency rate ( *n* = 5) was the most investigated main outcomes ( Table 1 ). For five techniques details on their clinical use was unavailable ( Table 1 ). Statistical power analysis and confounder adjustment were performed in none of the studies ( Table 1 ).

Seven studies proposed a novel sutureless anastomosis technique, with modifications such as use of absorbable ( *n* = 1) and nonabsorbable cuffs ( *n* = 1) in two studies. All studies were performed on animal subjects ( Table 1 ). Two studies were RCTs ( Table 1 ). Conventional interrupted suture technique was used in the control group in six studies and unabsorbable cuff technique in one study ( Table 1 ). The immediate postoperative patency rate ( *n* = 4) and anastomotic time ( *n* = 2) were the most investigated main outcomes ( Table 1 ). For five techniques details on their clinical use was unavailable and one technique was clinically used ( Table 1 ). Statistical power analysis and confounder adjustment were performed in none of the studies ( Table 1 ).

One RCT proposed a novel elliptic vesselotomy technique 4 on animals, which was clinically used ( Table 1 ). Statistical power analysis and confounder adjustment were not performed ( Table 1 ). One RCT proposed a modified lymphaticovenular anastomosis technique 5 on animals, with no statistical power analysis and confounder adjustment ( Table 1 ).

### Noncomparative Studies

Eighteen of the included studies were noncomparative studies ( Table 2 ). In total, 10 studies contained animal subjects ( *n* = 320) and 8 studies contained human subjects ( *n* = 173) ( Supplementary Appendix: Table S2 , available in the online version).

**Table 2:** Studies validating novel microsurgical techniques: noncomparative studies (16 studies)

Technique type	Study design (no. studies)	Type of subjects (no. studies)	Type of modification (no. studies)	Anastomosis type (no. studies)	Main outcome (no. studies)	Technique is clinically used (no. studies)	Power analysis performed (no. studies)	Confounder adjustment performed (no. studies)
Modified interrupted technique (5 studies) 34 35 36 37 38	Experimental (1) RCT (1) RO (1) CS (1) PO (1)	Animal (2) Human (3)	Posterior wall first (1) No turnover (1) Fewer sutures (1) Single loop (1) Temporary assisting suspension suture (1)	ETE (2) ETS (3)	Initial success rate (1) Postoperative PR (2) Postoperative FR (1) Complication rate (1) Operative time (1) Postoperative venous thrombosis (1)	Yes (1) N/A (4)	No (5)	No (5)
Modified continuous technique (1 study) 6	Experimental (1)	Animal (1)	Anterior and posterior wall sutures placed separately (1)	ETE (1)	Ischemic time (1)	N/A (1)	No (1)	No (1)
Modified sleeve anastomosis technique (2 studies) 39 40	Experimental (2)	Animal (2)	Heat-induced tissue welding (1) Suture placement (1)	ETE (2)	Postoperative PR (1) Postoperative FR (1)	N/A (2)	No (2)	No (2)
Modified vesselotomy technique (4 studies) 41 42 43 44	Experimental (1) RO (2) CS (1)	Animal (1) Human (3)	Longitudinal vesselotomy (1) V-shaped flap (1) Oblique transection (1) Diamond-shaped vesselotomy (1)	ETE (3) ETS (1)	Postoperative FR (1) Operative time (1) Vessel discrepancy ratio (1) Flap survival rate (1) Postoperative complication rate (1)	Yes (2) N/A (2)	No (4)	No (4)
Sutureless technique (4 studies) 45 46 47 48	Experimental (3) RCT (1)	Animal (4)	Heat-induced tissue welding (2) Unabsorbable cuff (2)	ETE (4)	Operative time (2) Postoperative PR (1) Postoperative FR (1) Postoperative subject survival (1)	No (2) N/A (2)	No (4)	No (4)
Modified lymphaticovenular anastomosis technique (2 studies) 49 50	PO (2)	Human (2)	Double barrel (1) Multiple barrel (1)	ETE (2)	Relief of lymphedema symptoms (2)	No (2)	No (2)	No (2)

Abbreviations: CS, case series; ETE, end-to-end; ETS, end-to-side; FR, flow rate; N/A, not available; PO, prospective observational; PR, patency rate; RCT, randomized controlled trial; RO, retrospective observational.

Five studies proposed a novel modified interrupted anastomosis technique, with modifications such as fewer sutures and single loop sutures ( Table 2 ). Two studies were performed on animal subjects and three on human subjects ( Table 2 ). One RCT, one prospective observational cohort study, and one retrospective observational cohort study were conducted ( Table 2 ). The immediate postoperative patency rate ( *n* = 2) was the most investigated main outcomes ( Table 2 ). One technique was clinically used, and for four techniques details on their clinical use was unavailable ( Table 2 ). Statistical power analysis and confounder adjustment were performed in none of the studies ( Table 2 ).

One experimental study proposed a novel modified continuous anastomosis technique 6 on animals ( Table 2 ). Statistical power analysis and confounder adjustment were not performed ( Table 1 ). Two experimental studies proposed modified sleeve anastomosis techniques on animals, with modifications such as heat-induced tissue welding ( Table 2 ). Statistical power analysis and confounder adjustment were not performed in both studies ( Table 2 ). Two prospective observational studies proposed multiple barrel modified lymphaticovenular anastomosis techniques on human subjects ( Table 2 ). As the main outcome, relief of lymphedema symptoms were measured ( Table 2 ). Statistical power analysis and confounder adjustment were not performed in both studies ( Table 2 ).

Four studies proposed a novel modified vesselotomy technique, with modifications such as longitudinal vesselotomy ( Table 2 ). One study was performed on animal subjects and three studies were performed on human subjects ( Table 2 ). Two techniques were clinically used, and for two techniques details on their clinical use was unavailable ( Table 2 ). Statistical power analysis and confounder adjustment were performed in none of the studies ( Table 2 ).

Four studies proposed a novel sutureless anastomosis technique, with modifications such as heat-induced tissue welding ( *n* = 2) and nonabsorbable cuffs ( *n* = 2) ( Table 2 ). All studies were performed on animal subjects ( Table 2 ). One study was an RCT ( Table 2 ). Operative time ( *n* = 2) was the most investigated main outcomes ( Table 2 ). For five techniques details on their clinical use was unavailable and one technique was clinically used ( Table 1 ). Statistical power analysis and confounder adjustment were performed in none of the studies ( Table 1 ).

## Discussion

### Summary of Findings

This systematic review summarized evidence from 48 articles, including data on both animal ( *n* = 2,318) and human ( *n* = 202) subjects. Thirty of the included studies were comparative studies, with 14 RCTs containing animal subjects and 1 RCT containing human subjects. Nine comparative studies validated novel modified interrupted suture techniques, six studies modified continuous techniques, six studies modified sleeve anastomosis techniques, one study a modified vesselotomy technique, seven studies sutureless techniques, and one study a modified lymphaticovenular anastomosis technique. Statistical power analysis and confounder adjustment were performed in one comparative animal study. Eighteen of the included studies were noncomparative studies, with two RCTs containing animal subjects. Five noncomparative studies validated novel modified interrupted suture techniques, one study a modified continuous technique, two studies modified sleeve anastomosis techniques, four studies modified vesselotomy techniques, four studies sutureless techniques, and two studies modified lymphaticovenular anastomosis techniques. Statistical power analysis and confounder adjustment were performed in none of the noncomparative animal or human studies.

### Current Methods of Technique Validation

Many types of validity are used in scientific literature. 7 Face validity, construct validity, and predictive validity are important aspects on which validity of microsurgical techniques should be based. First, face validity indicates if the new technique is as effective as the standard technique or not. Second, construct validity compares and correlates the current anastomosis outcomes with other outcomes, to reveal possible associations. At last, when these associations have been revealed, predictive validity can determine how the studied techniques will perform in clinical practice based on the used method of measurement. It remains abundantly clear from the studies assessed that while a plethora (hundreds) of reports is available detailing either a new trick, technique, or anastomosis modification, researchers and clinicians are completely unaware of the possibilities and responsibilities regarding proper validation. Even when techniques are validated, the exact facet of validity is not specifically mentioned, despite some articles being of high methodological rigor. Reviewers and editors should be wary of these practices and demand exact validation methodologies for the different facets of validity. Noncomparative studies should not be accepted as proper proof of validation. Best of all, predictive validity should be encouraged, that is, that a new trick of technique actually leads to better overall results in the clinical setting.

The noncomparative studies ( *n* = 16) included in this systematic review failed to properly validate their newly proposed techniques by including no control group, which makes their outcomes less reliable. No studies evaluated construct validity, except for Dindelegan et al, 3 which performed confounder adjustment. Besides, predictive validity has not been proven in any of the studies included, except for Dindelegan et al, 3 which is the most important and relevant type of validity in case of microsurgical techniques. Out of over 6,000 published studies about microsurgical techniques, only 47 proposed new techniques, of which only 1 study succeeded to properly validate their technique. The optimal situation of technique validation is when a technique is first validated in an RCT comparison in animals, and then for predictive validity in an RCT in a clinical setting. No study in our systematic review succeeded in reaching this standard. Therefore, the second-best strategy is to validate a technique in a prospective study/RCT clinically using a control group, worst case scenario historical matched controls, which is also not met by the included studies. At last, a fair method of validation is performing RCT comparison with conventional suture techniques in animals, which has been conducted in 14 studies included in this systematic review. 3 4 5 8 9 10 11 12 13 14 15 16 17 18 However, only one study among these RCTs succeeded in proper predictive validation, which has been mentioned earlier. 3 The validated techniques in these studies are merely modifications to existing anastomosis techniques or efforts to minimize steps in suturing anastomosis, aiming to reach a faster and more efficient anastomosis time, with the same or improved patency rate as conventional methods. Additional adhesive tools such as fibrin glue and cyanoacrylate were added to the anastomosis suturing steps to speed up the process of suturing and tricks, such as open guide suturing, were introduced to provide a better exposure during suturing and improve eversion of the vessel wall.

### Recommendations

Our results indicate that only very few studies, and even fewer RCTs have been conducted to validate new microsurgical techniques, the least of which in the patient population of interest. Although, these techniques are being routinely used in surgeries without proper evidence of their effectiveness or proof that they achieve their goal when used clinically. The current scientific methods employed in the validation of new microsurgical techniques have rudimentary and scientifically barren and need to be refined. Confounder adjustment with predefined confounders should be part of any data analysis plan, as well as adequate power analysis before commencing any study.

### Strengths and Limitations

To our knowledge, this article is the first systematic review discussing the current methods of validation of microsurgical techniques. Some limitations should be noted such as the fact that three studies did not define their main outcomes, which they simply address as, for example, “success rate.” Besides, the main outcomes measured in the studies appeared to be quite heterogeneous, which prevented us from pooling results or performing statistical inferences on the data collected. Lastly, 31 (67%) of the articles failed to mention whether their novel technique is being used in clinical practice. Articles should at least mention if their technique has been clinically used in their own institute, and if not, this information should also be reported.

## Conclusion

The current scientific methods employed in the validation of microsurgical techniques are rudimentary and should be reconsidered. Statistical analysis plans, including confounder adjustment and power analysis, should be employed routinely in each predefined statistical analysis plan.
