# Supplementary material for: Validation of Novel Microsurgical Vessel Anastomosis Techniques: A Systematic Review
Source: J Reconstr Microsurg. 2024 May 6;41(1):28–36. doi: 10.1055/a-2302-7126 (PMC11668557; doi:10.1055/a-2302-7126)
Supplement: Supplementary file 1 — Supplementary Material [file 10-1055-a-2302-7126-s23100230.pdf]

Supplementary Appendix: Table S1 Studies validating novel microsurgical techniques: comparative studies (30 studies)

Study (First author, year)	Study design	Country	Number of subjects	Name of technique validated	Type of technique	Description of technique	Main outcome (s)	Control group	Technique is clinically used	Power analysis performed	Confounder adjustment performed
Animal subjects											
Hou, 1987[19]	Comparative	USA	40 rats	Alternative interrupted technique	ETE	The first stitch is placed on the posterior wall of the vessel	Postoperative PR	Conventional interrupted suture technique	N/A	N	N
Siemionow, 1987[26]	Comparative	USA	160 rabbits	Asymmetrical vs. symmetrical adventitia sleeve technique	ETE	Asymmetrical sleeving of adventitia vs. Symmetrical trimming of the adventitia with collagen cuff wrapping vs. Symmetrical trimming of adventitia vs. Sleeve anastomosis technique	Postoperative PR	Arteries with adventitia and vessel preparation prior to anastomosis	N/A	N	N
Zhang, 1991[8]	RCT	China	40 rats	Sleeve anastomosis	ETE	One side cut and the placement of two sutures in separate vertical planes	Postoperative PR	Conventional interrupted suture technique	N/A	N	N
Saitoh, 1993[9]	RCT	Japan	29 rats	Loop sutures in sleeve anastomosis	ETE	Two loop sutures placed on the proximal stump of the vessel are passed through the wall of the distal vessel	Postoperative PR	Conventional interrupted suture technique	N/A	N	N
Adams Jr, 2000[4]	RCT	USA	104 rats	Elliptic hole, slit anastomosis	ETS	Elliptic vesselotomy with anastomosis in arteries and veins	Postoperative PR	Slit anastomosis in arteries and veins	Y	N	N
O'Neill, 2007[30]	Comparative	USA	60 rats	Photochemical tissue bonding technique (PTB)	ETE	PTB uses a combination of visible light and a photoreactive dye to create immediate bonds and a tight seal between tissue surfaces	Postoperative PR	Conventional interrupted suture technique	N	N	N
Cigna, 2008[10]	RCT	Italy	40 rats	Posterior wall first - Continuous interrupted - Airborne (PCA) technique	ETE	The first stitch is placed on the posterior wall of the vessel, the second and third sutures are placed very close to the first stitch, one above and one below it. The anastomosis is completed by using the Continuous-Interrupted technique	Postoperative PR	Continuous-Interrupted technique	N/A	N	N
Zhang, 2010[11]	RCT	China	40 rats	Modified interrupted suture technique	ETE	The modified technique entailed using fewer sutures (5–6 sutures) and fibrin glue	Postoperative PR	Conventional interrupted suture technique	Y	N	N

Supplementary Appendix: Table S1 (*Continued*)

Study (First author, year)	Study design	Country	Number of subjects	Name of technique validated	Type of technique	Description of technique	Main outcome (s)	Control group	Technique is clinically used	Power analysis performed	Confounder adjustment performed
Huang, 2011[12]	RCT	Germany	18 rats	Modified interrupted suture technique	ETS	The modified technique allowed for the compensation of size mismatches between donor and recipient vessels by placing a small incision from the edge of the recipient vessel	Postoperative PR	Conventional interrupted suture technique	N/A	N	N
Ishiura, 2017[5]	RCT	Japan	12 rats	Supermicrosurgical lymphaticovenular anastomosis	ETE	The largest selected lymphatic vessel was anastomosed to the recipient vein in an intima-to-intima coaptation manner	Postoperative PR	Lymphaticovenular implantation technique	N	N	N
Firsching, 1984[24]	Experimental	Germany	20 rats	Continuous-suture technique	ETE	Continuous-suture technique with resorbable suture material	Postoperative FR	Conventional interrupted suture technique	Y	N	N
Miyamoto, 2008[20]	Comparative	Japan	120 rats	Flow-through arterial anastomosis	ETE, ETS	ETE anastomoses were performed between the proximal and distal stumps of the axillary artery and the stumps of the common carotid artery	Postoperative FR	Conventional interrupted suture technique	N	N	N
Miyamoto, 2008[21]	Comparative	Japan	60 rats	Retrograde arterial anastomosis	ETE, ETS	The anastomosis is performed in an antegrade fashion	Postoperative FR, flap survival	Conventional interrupted suture technique	N	N	N
Euler, 1989[31]	Experimental	Germany	11 rats	Cuff technique	ETE	Nonsuture microsurgical vessel anastomosis using an absorbable cuff	Absorption rate	Unabsorbable cuff technique	N/A	N	N
Zhou, 2010[13]	RCT	Multi-country	80 mice	Cuff technique	ETE	Nonsuture microsurgical vessel anastomosis using a cuff	Surgical success rate	Conventional interrupted suture technique	N/A	N	N
Miyamoto, 2010[14]	RCT	Japan	45 rats	Intravascular stenting technique vs. open guide suture technique	ETE	Intravascular stenting technique: The first stay suture was placed on the side opposite the surgeon and tied. The stent was then inserted into both stumps, thereafter, anastomosis was completed as with the conventional technique<br>Open guide suture technique: Two interrupted sutures were placed on the anterior wall with untied method and tied in order.	Ischemia time, Postoperative PR	Conventional interrupted suture technique	N/A	N	N

(*Continued*)

 © 2024. The Author(s).

Supplementary Appendix: Table S1 (*Continued*)

Study (First author, year)	Study design	Country	Number of subjects	Name of technique validated	Type of technique	Description of technique	Main outcome (s)	Control group	Technique is clinically used	Power analysis performed	Confounder adjustment performed
Başar, 2012[15]	RCT	Turkey	36 rats	Continuous horizontal mattress suture technique	ETS	Between the two knots, on the front side first and then on the back side, four sutures were passed as horizontal mattress, then two sutures were tied with each other.	Anastomotic leakage, aneurysm formation, Postoperative FR	Conventional interrupted suture technique, Conventional continuous suture technique	N/A	N	N
Szabo, 2020[27]	Experimental	Hungary	60 chicken thighs<br>12 rats	Modified Lauritzen's sleeve-technique	ETE	Four sutures, including corner and pulling stitches, were added to the conventional sleeve anastomosis	Elongation, tensile strength, and elasticity of vessels	Conventional interrupted suture technique, Conventional continuous suture technique	N/A	N	N
Dindelegan, 2021[3]	RCT	Romania, The Netherlands	177 chicken thighs<br>43 rats	The double stitch everting technique	ETS	Leaving the needle inside the vessel wall in-between stitching	Suture symmetry score, Postoperative PR	Conventional interrupted suture technique	Y	Y	Y
Orădan, 2022[16]	RCT	Romania	24 rats	The use of cyanoacrylate in anastomosis	ETE	Anastomosis with three interrupted sutures with tissue adhesive being applied between the sutures to complete the anastomosis	Total operative time, anastomotic time, Postoperative FR	Conventional interrupted suture technique	Y	N	N
Lemaire, 2000[28]	Experimental	Canada	33 rats	Anastomosis with histoacryl glue and an intravascular soluble stent	ETE	The artery was prepared with histoacryl glue and an intervascular soluble stent was placed	Postoperative PR	Conventional interrupted suture technique	N/A	N	N
Le Hanneur, 2022[51]	Experimental	France	7 rats	Fibrin-glue-augmented sleeve anastomosis	ETE	Fibrin-based glue sleeve was added to conventional interruptured sutured anastomosis	Postoperative PR	Conventional interrupted suture technique	N	N	N
Mao, 2009[25]	Experimental	China	400 mice	Knotless technique	ETE	Knotless continuous suture	Postoperative anastomosis bleeding and stenosis	Conventional continuous suture technique	N/A	N	N
Maitz, 1999[32]	Comparative	Austria	90 rats	Biodegradable laser-activated solid protein solder	ETE	Sutureless microvascular anastomoses by a biodegradable laser-activated solid protein solder	Postoperative PR	Conventional interrupted suture technique	N/A	N	N
Riggio, 1999[29]	Experimental	Italy	81 rats	Modified sleeve technique	ETE	A vertical cut was placed in the sleeve (invagination)	Postoperative PR	Conventional interrupted suture technique	N/A	N	N
Sacak, 2013[33]	Experimental	Turkey	64 rats	Fibrin glue and venous cuff technique	ETE	Anastomosis was created with fibrin glue, a venous cuff was added to the anastomosis site	Postoperative PR	Conventional interrupted suture technique	N/A	N	N
Marni, 1996[17]	RCT	Italy	20 rats	Modified continuous technique	ETS		Postoperative PR		N/A	N	N

 © 2024. The Author(s).

Supplementary Appendix: Table S1 (*Continued*)

Study (First author, year)	Study design	Country	Number of subjects	Name of technique validated	Type of technique	Description of technique	Main outcome (s)	Control group	Technique is clinically used	Power analysis performed	Confounder adjustment performed
						Last stitches are placed from the inside to the outside		Conventional interrupted suture technique			
Ariyakhagorn, 2009[18]	RCT	Germany	72 rats	Knotless technique	ETE	The last stitch is made as close as possible to the starting stitch. The two starting and finishing filaments of the suture are divided without making a knot.	Anhepatic time, graft and subject survival	Conventional interrupted suture technique	N/A	N	N
Human subjects											
Odobescu, 2014[22]	RCT	Canada	20 cadavers	Interrupted horizontal mattress sutures	ETE	The anastomosis started with a backhand pass of the horizontal mattress suture and returned with the forehand pass approximately 1 mm apart, such that the center of the suture would coincide with the zenith of the vessel	Anastomotic leakage and time	Conventional interrupted suture technique	N/A	N	N
Miyagi, 2008[23]	RO	Japan	9 patients	Back wall support suture technique	ETE	Two sutures were placed at the deepest, most difficult points in the hepatic artery for backside support. Each stitch was placed from the inner side of the arterial wall to the outer side with double needle sutures. The subsequent sutures were placed forward on either side adjacent to the previous suture	Rate of hepatic artery thrombosis	Conventional interrupted suture technique	N/A	N	N

Abbreviations: ETE, end-to-end; ETS, end-to-side; N, no; N/A, not available; Postoperative FR, postoperative flow rate; Postoperative PR, postoperative patency rate; RCT, randomized controlled trial; RO, retrospective observational; Y, yes.

 © 2024. The Author(s).

Supplementary Appendix: Table S2 Studies validating novel microsurgical techniques: noncomparative studies (16 studies)

Study (First author, year)	Study design	Country	Number of subjects	Name of technique validated	Type of technique	Description technique	Main outcome(s)	Technique is clinically used	Power analysis performed	Confounder adjustment performed
Animal subjects										
Orbay, 1985 [41]	Experimental	Switzerland	10 rats	Elongated and slit-like anastomosis	ETE	The anastomosis is facilitated by a longitudinally cut vein	Postoperative FR	N/A	N	N
Duarte, 1987[39]	Experimental	USA	72 rats	Thermic sleeve anastomosis	ETE	Proximal and distal stump of the vessel have been intussuscepted and are welded on two spots	Postoperative PR	N/A	N	N
Schubert, 2004[45]	RCT	Austria	42 rats	Bipolar anastomosis technique	ETE	Heat-induced tissue welding is used to create the anastomosis	Operative time, Postoperative PR	N/A	N	N
Ulusal, 2005[34]	RCT	Taiwan	20 mice	Temporary assisting suspension suture technique	ETE	Placement of suspension loose sutures	Initial success rate	N/A	N	N
Hudson, 1998[6]	Experimental	South Africa	48 rats	Modified continuous anastomosis technique	ETE	The posterior wall and anterior wall continuous sutures are placed separately after two separate stay sutures. A closing suture is placed when flow through the anastomosis is confirmed	Ischemic time	N/A	N	N
Lauritzen, 1978[40]	Experimental	Sweden	20 rats	Sleeve anastomosis technique	ETE	Two starting sutures are placed and the proximal vessel is placed inside the lumen of the distal vessel	Postoperative FR, postoperative PR	N/A	N	N
Holmin, 1983[35]	Experimental	Sweden	20 rats	Portacaval shunt	ETS	A simplified method of ETS anastomosis with three sutures	Postoperative PR	N/A	N	N
Savas, 1985[46]	Experimental	UK	40 rats	Sutureless cuff technique	ETE	A cuff was created from vessel lumps and connected together without sutures	Postoperative PR, postoperative subject survival	N/A	N	N
Fensterer, 2014[47]	Experimental	USA	8 rats	Cuff placement technique	ETE	Facilitating vessel eversion by the creation of a wedge-shaped gap to generate an adjustable cuff diameter at one end of the cuff and the creation of a barb to facilitate the fixing of draped vessel edges to the cuff.	Operative time	N	N	N
Schubert, 2006[48]	Experimental	Austria	40 chickens	Bipolar anastomosis technique (BAT)	ETE	Anastomosis is created by heat-induced tissue-welding	Postoperative PR and FR	N	N	N
Human subjects										
Bakhach, 2015[42]	CS	Lebanon	14 patients	V-plasty technique	ETE	Creating a V-shaped flap in the vessel of larger diameter	Vessel discrepancy ratio	Y	N	N
Inbal, 2019[43]	RO	USA	100 patients	Modified Kunlin's technique	ETE	An oblique transection of the donor and recipient vessels is performed with greater angle of transection for the smaller vessel to approximate circumferences	Flap survival rate, Postoperative complication rate	Y	N	N
Yamamoto, 1999[36]	CS	Japan	17 patients	Back wall technique	ETE	The back wall is sutured first	Postoperative FR and complication rate	N/A	N	N

Supplementary Appendix: Table S2 (*Continued*)

Study (First author, year)	Study design	Country	Number of subjects	Name of technique validated	Type of technique	Description technique	Main outcome(s)	Technique is clinically used	Power analysis performed	Confounder adjustment performed
Masoodi, 2022[49]	PO	Austria	12 patients	Double barrel lymphaticovenous technique	ETE	Two lymphatic vessels have been intussuscepted into the vein	Relief of lymphedema symptoms	N	N	N
Nakagawa, 2008[37]	RO	Japan	9 patients	Modified technique for anastomosis of the posterior wall	ETS	Short pedicle without turn-over to the posterior wall	Postoperative venous thrombosis	N/A	N	N
Sen, 2006[44]	RO	Turkey	5 patients	Diamond arteriotomy technique	ETS	Diamond-shaped arteriotomy is performed	Operative time	N/A	N	N
Matsuo, 2020[38]	PO	Japan	7 patients	Single loop interrupted suture technique	ETS	Knots were placed after loop stitch placement	Postoperative PR, operative time	Y	N	N
Chen, 2015[50]	PO	Japan	9 patients	Octopus lymphaticovenular technique	ETE	Multiple lymphatic vessels have been intussuscepted into the vein	Relief of lymphedema symptoms	N	N	N

Abbreviations: CS, case series; ETE, end-to-end; ETS, end-to-side; N, no; N/A, not available; PO, prospective observational; Postoperative FR, postoperative flow rate; Postoperative PR, postoperative patency rate; RCT, randomized controlled trial; RO, retrospective observational; Y, yes.

 © 2024. The Author(s).
